# A diffusion tensor imaging-based multidimensional study of brain structural changes after long-term high-altitude exposure and their relationships with cognitive function

**DOI:** 10.3389/fphys.2024.1487953

**Published:** 2024-11-13

**Authors:** Ning Liu, Li Feng, Shuangwei Chai, Hailong Li, Yuanyuan He, Yongyue Guo, Xin Hu, Hengyan Li, Xiangwei Li, Zan Zhou, Xiaomei Li, Yonghong Huang, Wanlin He, Xiaoqi Huang, Yunhong Wu, Jinli Meng

**Affiliations:** ^1^ Department of Radiology, Hospital of Chengdu Office of People’s Government of Tibetan Autonomous Region (Hospital. C.T.), Chengdu, Sichuan, China; ^2^ Department of Radiology and Huaxi MR Research Center (HMRRC), Functional and Molecular Imaging Key Laboratory of Sichuan Province, West China Hospital, Sichuan University, Chengdu, Sichuan, China; ^3^ Research Unit of Psychoradiology, Chinese Academy of Medical Sciences, Chengdu, Sichuan, China; ^4^ Department of Endocrinology and Metabolism, Hospital of Chengdu Office of People’s Government of Tibetan Autonomous Region (Hospital. C.T.), Chengdu, Sichuan, China

**Keywords:** DTI, TBSS, long-term adaptation, high-altitude, cognitive function

## Abstract

**Background:**

Brain structure changes after long-term adaptation to the high-altitude environment; however, related studies are few, results are in consistent, and long-term effects on cognitive function and pathophysiological mechanisms are unclear. Therefore, diffusion tensor imaging (DTI) was used to investigate the damage to white matter fiber tracts and correlations between brain structural abnormalities and cognitive function.

**Methods:**

Forty healthy Han people living on the high-altitude and 40 healthy Han people living on the plains were enrolled in this study and underwent magnetic resonance imaging, emotional state assessment, and cognitive function tests. The sex, age, education level, and social status of the two groups were not different. The tract-based spatial statistics (TBSS) method was used to analyze the DTI parameters of the white matter fiber tracts of the two groups. Moreover, the partial correlation method (age and sex as covariates) was used to analyze the correlations between the intergroup differences in the DTI parameters and a series of clinical indicators of emotional state and cognitive function. Two-sample t tests, Mann-Whitney *U* test, generalized linear model, or chi-square tests were used for statistical analysis.

**Results:**

Compared with those individuals in the plain group, the scores on the PSQI, SDS, SAS, PHQ-9, and GAD-7 of individuals in the high-altitude group were higher, while the scores on the DST-Backwards, MoCA, and MMSE in the high-altitude group were lower. The fractional anisotropy (FA) value of the body of the corpus callosum in the high-altitude group was lower than that in the plain group. The FA value of the body of the corpus callosum in the plain group was negatively correlated with the Logical Memory, while no significant correlation was found in the high-altitude group.

**Conclusion:**

This study revealed that long-term exposure to a high-altitude environment could lead to a series of changes in sleep, emotion, and cognitive function and irreversible damage to the white matter microstructure of the body of the corpus callosum, which is the related brain region responsible for logical memory. The absence of logical memory impairment in the healthy Han Chinese population living on the high-altitude in this study may be due to the existence of adaptive compensation after long-term high-altitude exposure.

## 1 Introduction

Globally, the high-altitude terrain covers a wide area. In China, more than 10 million people permanently live on the Qinghai-Tibetan plateau (QTP) at an altitude of 2,200 m or more (Wu and Kayser, 2006). In addition, thousands of people migrate from the plains to high-altitude areas every year for altitude training (Lundby and Robach, 2016), work or study. The impact of the unique natural conditions of the high-altitude (hypoxia, low pressure, low temperature, and strong ultraviolet (UV) rays) on human health has attracted increasing attention in the medical community. As the altitude increases, the atmospheric pressure and partial pressure of oxygen decrease, and the oxygen supply to tissues and organs decreases. Short-term high-altitude exposure can cause adaptive changes in multiple organs and multiple systems, especially the central nervous system, which manifest as cognitive decline (Henig and Pierson, 2000; Zhang Y. Q. et al., 2022), abnormal mood (Figueiredo et al., 2022), and poor sleep quality (de Aquino Lemos et al., 2012; Morrison et al., 2017). After long-term high-altitude exposure, hypoxia can directly stimulate brain tissue, causing nonadaptive chronic damage to the structure of the brain (Luo et al., 2023); at the same time, physiological adaptations of other systems (such as the circulatory and respiratory systems) can further cause the accumulation of brain damage through feedback mechanisms, which may eventually lead to long-term irreversible brain damage (Nation et al., 2017), thus causing dysfunctions (Hayashi et al., 2005; An et al., 2022) other than cognitive dysfunction (Dietz and McKiel, 2000; Ma et al., 2019; Zhang Z. A. et al., 2022). Longer exposure to low-pressure and hypoxic environments is associated with more severe brain injury (Chen et al., 2023), accompanied by structural changes (Terraneo and Samaja, 2017; Hoiland et al., 2018) in the brain.

Magnetic resonance imaging (MRI) is a widely used noninvasive neuroimaging technique that can effectively reveal brain structure, and MRI-based analysis of the effects of a high-altitude environment on brain structure can reveal the compensatory process and damage mechanism of the brain in a high-altitude environment. Previous studies have revealed that brain structural changes caused by a high-altitude environment manifest mainly as cortical atrophy and periventricular white matter hyperintensity on MRI (Garrido et al., 1996). At present, diffusion tensor imaging (DTI) technology has been used in studies of white matter microstructure, and the tract-based spatial statistics (TBSS) method has been used to measure the fractional anisotropy (FA), axial diffusivity (AD), radial diffusivity (RD), and mean diffusivity (MD) of the white matter fibers, all of which can be used to quantify white matter microstructure. Related studies have shown that a high-altitude environment can lead to decreased FA values in bilateral corticospinal tracts and dorsal midbrain reticular formations (Zhang et al., 2012), bilateral corona radiata and left internal capsule (Zhang Y. Q. et al., 2022), the left middle cerebellar peduncle (Zhang et al., 2012), the left superior longitudinal fascicle (Zhang et al., 2010; Zhang et al., 2012; Chen et al., 2019), the left optic radiation area (Zhang et al., 2010), right posterior cingulum fibers (Yan et al., 2010; Zhang et al., 2012), the right prefrontal cortex (Yan et al., 2010), the right anterior limb of the internal capsule (Chen et al., 2019), the genu of the corpus callosum (Zhang Y. Q. et al., 2022), the body of the corpus callosum (Zhang et al., 2012; Chen et al., 2019), and the splenium of the corpus callosum (Zhang et al., 2012). Studies have also shown that a high-altitude environment can increase the FA value in some brain regions (including the corpus callosum) (Zhang et al., 2010; Zhang et al., 2013; Chen et al., 2019), and increased FA values are positively correlated with visuospatial scores (McGuire et al., 2016). Chen et al. (2017) showed that after high-altitude exposure, FA values increased and MD values decreased in some brain nuclei (putamen, globus pallidus, caudate nucleus, dentate nucleus, red nucleus, and substantia nigra), but FA and MD values were linearly correlated with the iron concentration only in the putamen. These studies focused mainly on populations with short-term high-altitude exposure. There are few studies on brain structural changes after long-term adaptation to a high-altitude environment, the obtained results are inconsistent, and the pathophysiological mechanisms remain unclear. Therefore, this study focused on changes in the white matter microstructure of the brain after long-term adaptation to a high-altitude environment.

Changes in brain structure caused by a high-altitude environment are significantly correlated with cognitive function. In most previous studies, a single indicator of cognitive function, sleep or emotion was used for the correlation analysis. There are few studies on the multidimensional correlations between brain structural changes and cognitive function caused by long-term adaptation to a high-altitude environment (2,616–4,200 m). Therefore, the objective of this study was to use DTI to investigate the damage to white matter fiber tracts in a population after long-term adaptation to a high-altitude environment, to further explore the correlations between brain structural abnormalities and multiple clinical cognitive and physiological indicators, and to elucidate the brain structural changes and cognitive impairment characteristics after long-term adaptation to a high-altitude environment in hopes of providing a clinical reference for early intervention measures for the brain changes caused by hypoxia.

## 2 Materials and methods

### 2.1 Participants

**FIGURE 1:**
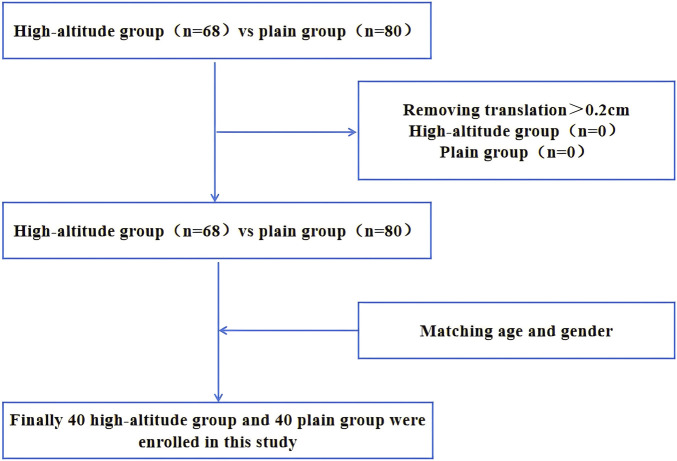
Flow chart of participant inclusion in the analysis.

All participants were Han Chinese. Subjects in the high-altitude group have lived on the high-altitude environment for at least 10 years and are partially permanently lived in high-altitude, and subjects in the plain group are permanently lived in the plains. The inclusion criteria were as follows: (1) no neurological diseases; (2) no contraindication for MRI examination; and (3) no history of tumor or severe metabolic disease. The exclusion criteria were as follows: (1) a known history of brain injury, epilepsy, stroke, alcohol or other substance dependence, Parkinson’s disease, mood disorder or other diseases that may affect cognitive function, or major diseases (such as cancer); and (2) patients with contraindications for MRI scanning. We ultimately recruited 40 healthy individuals living in a high-altitude habitat (female/male: 23/17; mean age: 48.15 ± 6.95) and 40 healthy individuals living in a plain habitat (female/male: 21/19; mean age: 45.20 ± 6.50) in the study (Figure 1). The baseline data of all participants were collected, including demographic data, clinical information (weight, height, blood pressure, and body mass index) and physiological and biochemical indicators (lipid indicators, complete blood count, etc.). The sex, age, education level, and social status of the two groups were not different.

### 2.2 Neuropsychological assessments

Ten neuropsychological scales, including the Pittsburgh Sleep Quality Index (PSQI), Self-rating Anxiety Scale (SAS), Self-Rating Depression Scale (SDS), Patient Health Questionnaire-9 (PHQ-9), Generalized Anxiety Disorder-7 (GAD-7), Mini-Mental State Examination (MMSE), Montreal Cognitive Assessment (MoCA), Digital Span Test (DST) Forwards/Backwards, Logical Memory Immediately/Delay, and Visual Memory Immediately/Delay, were used to assess cerebral function in all participants.

### 2.3 Image acquisition

T1-weighted structural MRI and DTI scans were obtained on a 3.0 T MRI scanner (Philips Achieva, Chengdu, China) equipped with an 8-channel head coil. A T1-weighted structural MRI scan was acquired via the following scan parameters: repetition time (TR) = 8.1 ms; echo time (TE) = 3.7 ms; voxel size = 1 × 1 × 1 mm³; field of view (FOV) = 256 × 256 mm; slice thickness = 1 mm; and flip angle = 12°. As a result of 33 noncollinear directions with a 1,000 s/mm^2^ b-factor and 1 repetition with no diffusion weight, diffusion-weighted images were acquired via the following scan parameters: TR = 7,150 m; TE = 105 m; voxel size = 2.2 × 2.2 × 2.2 mm³; FOV = 212 × 212 mm; and slice thickness = 2.2 mm.

### 2.4 Statistical analysis

The analyses of the diffusion data are based on a voxel-wise statistical method using TBSS (Smith et al., 2006) in FSL (Smith et al., 2004)^.^ To generate a mean FA tract skeleton for each participant, TBSS applies voxel-wise cross-subject statistics to all the FA data. This method focuses on the centers of all-fiber bundles that are common to all participants, and each FA image is aligned and affined in a 1 × 1 × 1 mm MNI152 space. All the FA images are subsequently averaged to create a mean FA image. The mean FA is entered into the generated tract skeleton that contains the centers of the tracts, which are required to generate the tract skeletons for all groups. By filling in the skeleton with maximal FA values obtained from the nearest tract center, a skeletonized FA map is generated from the aligned FA images for every subject. The target images are aligned via affine MNI152 space. Two-group difference analysis (adjusted for covariates) was used to assess differences between the high-altitude group and the plain group with voxel-wise cross-grouping in all voxels with an FA of ≥0.25. The t statistics maps are generated via the Monte Carlo permutation test (5,000 permutations). T statistical maps between the groups are calculated using thresholds for statistical images with a *p* < 0.05 and family-wise error correction for multiple voxel comparisons. Finally, we obtained cluster and peak information by randomizing for each of the previous comparisons and extracting significant *p* value areas in the MNI152 standard space. We also performed the same analysis on other DTI parameters (MD, RD and AD). Additionally, to investigate the relationship between structural brain alterations and cognitive function, we performed a partial correlation analysis of the extracted significant cluster with the neuropsychological scale scores (covariates: age and sex). When the clinical indicators, emotional state assessments, and other indicators were compared between the groups, the quantitative data were analyzed via a two-sample *t*-test or Mann-Whitney *U* test, while the chi-square test was used to compare qualitative data. The generalized linear model (PSQI, SAS, SDS, PHQ-9, and GAD-7 as covariates) was applied for the between-group comparison of cognitive function scores. Differences were considered statistically significant when the two-sided p was <0.05.

## 3 Results

The characteristics of the healthy individuals living in a high-altitude habitat and healthy individuals living in a plain habitat are shown (Table 1).

**TABLE 1:** Demographical results and neuropsychological characteristics in high-altitude habitation health controls and plain habitation health controls.

Characteristics	HAG		PG		Significance	
	Available data (n)	Mean ± SD/median (IQR)	Available data (n)	Mean ± SD/median (IOR)	t/z/X^2^-Value	*p*-value
Age [years]	40	48.15 ± 6.95	40	45.20 ± 6.50	1.962	0.053
Gender (female/male)	23/17	——	21/19	——	0.202	0.653
Education level [years]	6	12 ± 5.83	10	16.1 ± 4.41	−1.600	0.132
Social status	35	——	35	——	5.393	0.145
Enterprises and institutions personnel	24	——	16	——	——	——
Health technicians	10	——	19	——	——	——
Unemployed person	1	——	0	——	——	——
BMI [kg/m^2^]	36	23.92 ± 4.44	37	22.44 ± 2.29	1.783	0.080
SBP [mmHg]	31	116.87 ± 11.79	27	115.74 ± 12.67	0.352	0.726
DBP [mmHg]	31	78.52 ± 13.40	27	72.78 ± 10.10	1.819	0.074
Smoke (Yes/No) [years]	37 (16/21)	——	37 (6/31)	——	6.469	0.011*
Drink (Yes/No) [years]	37 (17/20)	——	37 (13/24)	——	0.897	0.344
RBC [10^12^/L]	23	4.56 ± 0.54	24	4.79 ± 0.55	−1.451	0.154
HGB [g/L]	23	140.48 ± 16.25	24	142.88 ± 15.20	−0.522	0.604
HCT [%]	23	43.19 ± 5.20	24	43.43 ± 4.54	−0.170	0.865
MCV [fL]	23	94.86 ± 3.84	24	91.22 ± 6.64	2.290	0.027*
MCH [pg]	23	29.68 ± 6.01	24	29.95 ± 2.14	−0.205	0.838
MCHC [g/L]	23	325.61 ± 10.25	24	328.54 ± 9.82	−1.001	0.322
HbA1c [%]	4	5.93 ± 0.46	7	6.00 ± 0.52	0.727	0.817
TG [mmol/L]	5	1.59 ± 1.30	12	1.53 ± 0.60	0.132	0.897
HDL [mmol/L]	17	1.29 ± 0.38	12	1.49 ± 0.39	−1.408	0.171
LDL [mmol/L]	17	2.78 ± 1.02	12	3.06 ± 0.67	−0.836	0.410
VLDL [mmol/L]	17	0.55 ± 0.43	12	0.47 ± 0.17	0.100	0.523
Neuropsychological Characteristics						
PSQI	31	9.23 ± 4.39	35	5.14 ± 3.07	4.328	0.000*
SDS	32	27.50 (11.00)	36	22.00 (6.00)	−3.526	0.000*
Neuropsychological Characteristics						
SDS-Standard	32	34.50 (13.00)	36	28.00 (8.00)	−3.526	0.000*
SAS	32	28.50 (10.00)	36	23.50 (8.00)	−3.113	0.002*
SAS-Standard	32	35.50 (12.00)	36	29.00 (10.00)	−3.113	0.002*
PHQ-9	32	5.50 (6.00)	36	0.00 (3.00)	−4.353	0.000*
GAD-7	32	4.00 (7.00)	36	0.00 (3.00)	−2.973	0.003*
DST-Forwards	32	8.00 (2.00)	36	9.00 (2.00)	3.828	0.050
DST-Backwards	32	4.00 (1.00)	36	5.00 (2.00)	15.485	0.000*
MoCA	32	27.00 (4.00)	31	28.00 (3.00)	7.578	0.006*
MMSE	30	29.00 (2.00)	31	30.00 (1.00)	10.940	0.001*
Logical Memory-Immediately	27	12.85 ± 3.54	34	13.41 ± 3.95	0.115	0.734
Logical Memory-Delay	26	12.38 ± 3.98	33	12.48 ± 3.81	0.003	0.959
Visual Memory-Immediately	24	7.50 (4.00)	30	6.00 (4.00)	0.043	0.836
Visual Memory-Delay	23	8.00 (3.00)	29	8.00 (5.00)	0.013	0.909

Notes: *: *p* < 0.05. HAG, high-altitude group; PG, plain group; IQR, interquartile range; BMI, body mass index; SBP, systolic blood pressure; DBP, diastolic blood pressure; RBC, red blood corpuscle; HGB, hemoglobin; HCT, hemoglobin; MCV, mean corpuscular volume; MCH, mean corpsular hemoglobin; MCHC, mean corpsular hemoglobin concentration; HbA1c, glycosylated hemoglobin; TG, triglyceride; HDL, high density lipoprotein; LDL, low density lipoprotein; VLDL, very low density lipoprotein; PSQI, pittsburgh sleep quality index; SDS, the Self-rating Depression Scale; SDS-Standard, the standard score on Self-rating Depression Scale; SAS, the Self-rating Anxiety Scale; SAS-Standard, the standard score on Self-rating Anxiety Scale; PHQ-9, the Patient Health Questionnaire-9; GAD-7, Generalized Anxiety Disorder-7; DST, digital span test; MoCA, montreal cognitive assessment; MMSE, the Mini-Mental State Examination.

**FIGURE 2:**
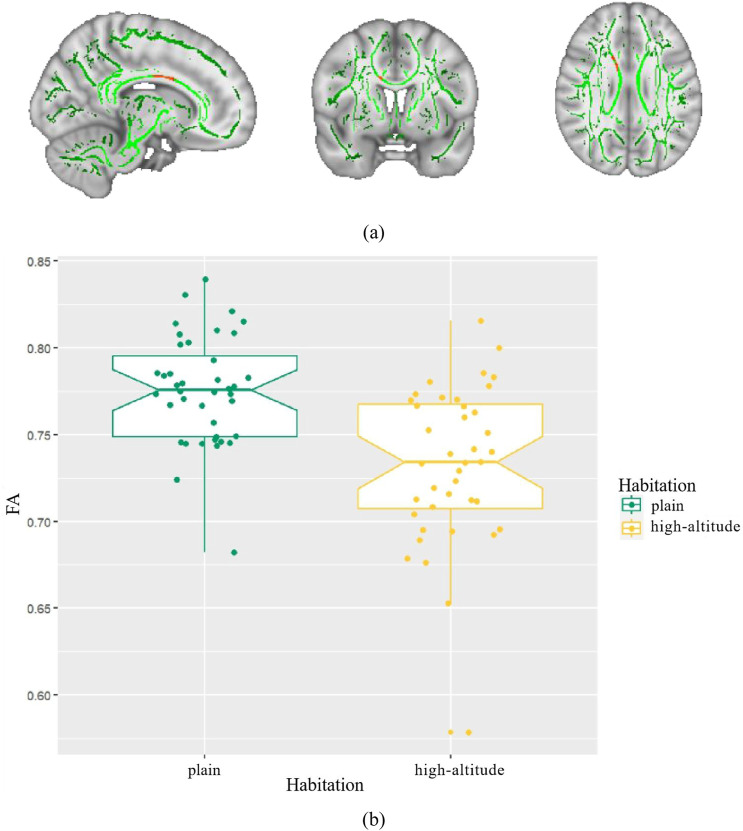
Differences in fractional anisotropy (FA) between high-altitude group and plain group. Notes: **(A)** The mean FA skeleton across all subjects is shown in green over the FMRIB58-FA-1mm template; **(B)** Boxplot of mean FA in high-altitude group, plain group.

**FIGURE 3:**
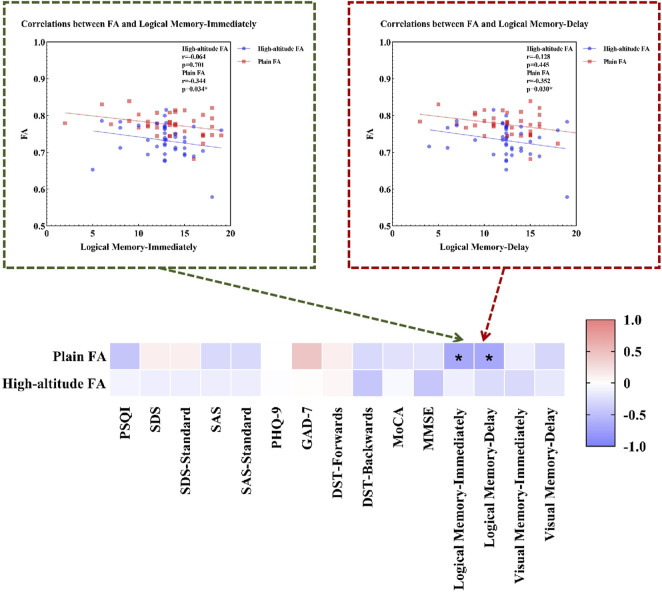
Correlations between FA and neuropsychological characteristics. Notes: Plain: plain group; High-altitude, high-altitude group; FA, fractional anisotropy; PSQI, Pittsburgh Sleep Quality Index; SDS, the Self-rating Depression Scale; SDS-Standard, the standard score on Self-rating Depression Scale; SAS, the Self-rating Anxiety Scale; SAS-Standard, the standard score on Self-rating Anxiety Scale; PHQ-9, the Patient Health Questionnaire-9; GAD-7, Generalized Anxiety Disorder-7; DST, Digital Span Test; MoCA, Montreal Cognitive Assessment; MMSE, the Mini-Mental State Examination.

We found that healthy individuals living in a high-altitude habitat had lower FA values in the body of the corpus callosum than healthy individuals living in a plain habitat (*p* < 0.05), but no significant between-group differences were observed in the other diffusion parameters (Figure 2). The coordinates of all the extracted areas as well as the *p*-values are reported (Table 2). Furthermore, in healthy individuals living in a plain habitat, FA values in the body of the corpus callosum were negatively correlated with both the Logical Memory-Immediately score (*p* = 0.034, r = −0.344) and the Logical Memory-Delay score (*p* = 0.030, r = −0.352). However, no significant correlation was found in healthy individuals living in a high-altitude habitat (Figure 3).

**TABLE 2:** White matter region of lower FA in high-altitude group compared with plain group.

Index	Tract label	Voxels	MNI-space			*p*-value
			x	y	z	FWE corrected
1	Body of corpus callosum	270	12	2	29	0.034

Notes: FA, fractional anisotropy; FWE, family-wise error; MNI, montreal neurological institute.

## 4 Discussion

This study investigated the DTI-based brain white matter fiber bundle of healthy Han people living on the high-altitude (high-altitude group) and healthy Han people living in the plains (plain group) to provide multidimensional clinical data as a reference for the study of brain structural alterations and cognitive function after long-term high-altitude exposure. This study revealed the following:

1) The significant intergroup differences between the high-altitude group and the plain group were mainly in emotional and cognitive function indicators, which may be due to the influence of the high-altitude environment. The anxiety, depression and cognitive abilities of the high-altitude group were worse than those of the plain group.
2) This study found that the FA value in the body of the corpus callosum in the high-altitude group was lower than that in the plain group, but no significant between-group differences were observed in other DTI parameters, suggesting that the corpus callosum is sensitive to the high-altitude environment and susceptible to the influence of hypoxia, which causes irreversible damage to the white matter microstructure.
3) Correlation analysis revealed that the FA value of the body of the corpus callosum in the plain group was negatively correlated with the Logical Memory-Immediately and Logical Memory-Delay scores; however, no significant correlation was found in the high-altitude group, indicating that the corpus callosum is responsible for logical memory. Due to adaptive compensation after the long-term high-altitude exposure, the logical memory function impairment does not occur in healthy Han people living on the high-altitude.

This study revealed that, compared with the plain group, the high-altitude group had increased PSQI, SDS, SAS, PHQ-9, and GAD-7 scores and decreased DST-Backwards, MoCA, and MMSE scores, which was consistent with previous study results. Hornbein et al. (1989) reported slight decreases in language and visual long-term memory and increased error rates in aphasia screening tests in climbers exposed to ultrahigh altitudes. Cognitive function declines significantly within one to 2 weeks of being in a high-altitude environment, and symptoms, such as memory loss, behavior changes, and slow thinking, occur. With prolonged exposure, adaptation to the hypoxic conditions of the high-altitude initially occurs, and the cognitive function of individuals recovers slightly, but full recovery to preexposure levels is difficult (Ling-Ling and Ming, 2017). Long-term high-altitude exposure can cause cognitive impairment, manifested as decreased inhibitory control, attention, and memory (Li and Wang, 2022). This finding may be due to a series of compensatory responses to maintain homeostasis produced by the body in the unique low-pressure and hypoxic high-altitude environment (Quillinan et al., 2016). As the control center of the human body, the brain is the most sensitive to hypoxia. During acute hypoxia, the body initiates the systemic cardiopulmonary reflex to compensate, resulting in vasodilation and hyperventilation (Sharp and Bernaudin, 2004) and causing dizziness, headache, shortness of breath and fatigue. During long-term hypoxia, the liver and kidneys secrete large amounts of erythropoietin, which increases the hemoglobin concentration and packed cell volume (PCV), resulting in an increase in erythrocytes and causing high-altitude polycythemia (HAPC), which can negatively affect the cognitive function of the individual (Li and Wang, 2022). Cognitive abnormalities are likely to occur in a high-altitude environment, which may be closely related to mechanisms such as oxidative stress, neurotransmitter or neuronal cell damage, the involvement of hypoxia-inducible factors, and inflammation (Chen et al., 2023). In general, in a high-altitude environment, cognitive function first decreases, then moderately increases, and finally decreases, resulting in cognitive function impairment (An et al., 2017). In addition, being in a high-altitude environment affects sleep quality (de Aquino Lemos et al., 2012; Morrison et al., 2017). Kong et al. (2011) revealed that, in a high-altitude environment, the sleep quality of soldiers with polycythemia was worse than that of healthy soldiers, which could further lead to abnormal mood and exacerbate irritability and depression (Dewald et al., 2010; Rosenzweig et al., 2015), and this effect was more evident with increasing altitude (Figueiredo et al., 2022). In summary, body damage caused by a high-altitude environment is related to altitude and duration of exposure, i.e., the higher the altitude and the longer the exposure time, the greater the injury.

This study revealed that the FA value of the body of the corpus callosum in the high-altitude group was lower than that in the plain group. The corpus callosum is located at the bottom of the interhemispheric fissure; is the largest white matter fiber tract in the central nervous system; is the most important hub between the cortical areas of the left and right cerebral hemispheres; connects the fibers of the motor, sensory and visual cortices (von Richthofen et al., 2003); and is closely related to emotion, cognitive function, movement, and vision. The corpus callosum is sensitive to a high-altitude environment and easily senses changes in the partial pressure of oxygen, causing different degrees of damage. Humans who acutely enter high-altitudes develop transient bilateral visual loss associated with cytotoxic damage to the corpus callosum (Yang et al., 2023). Patients with high-altitude cerebral edema present with multiple microbleeds along the corpus callosum (Schommer et al., 2013; Son et al., 2021; Karki et al., 2022) and hemosiderin deposition (Kallenberg et al., 2008; Schommer et al., 2013). Prolonged high-altitude exposure may damage blood vessels and promote neuronal apoptosis and the abnormal expression of related proteins (Cramer et al., 2019; Cao et al., 2023), thereby causing white matter microstructural damage. Zhang Y. Q. et al. (2022) showed that the FA value of the corpus callosum (body, splenium) decreased after short-term mountain climbing, which is consistent with the results of this study. In addition, after 2 years of high-altitude exposure, the FA value of the body of the corpus callosum decreases, and the FA value of the genu of the corpus callosum increases (Chen et al., 2019). After long-term adaptation to a high-altitude environment, the FA value of the corpus callosum increases (Zhang et al., 2010). The FA value is the diameter and density of white matter fibers and reflects the microscopic structural characteristics of white matter fibers (Chen et al., 2016). A decrease in the FA value indicates that white matter microstructural integrity is damaged (Basser and Pierpaoli, 1996), which is associated with local brain edema, negative changes to the cerebrospinal fluid (CSF), damage to the myelin sheath structure, changes in axonal morphology and structure, and changes in the interaxial spacing of fiber tracts (Beaulieu, 2002). In this study, a decrease in the FA value indicated that long-term adaptation to a high-altitude environment caused irreversible damage to the white matter microstructure of the corpus callosum. White matter structure is also disrupted with age (Cao et al., 2021), and white matter destruction can lead to hippocampal atrophy and induce cognitive impairment and dementia (Alber et al., 2019; Celle et al., 2021; Reas et al., 2021). Brain structural changes caused by long-term exposure to a high-altitude environment constitute the anatomical basis of cognitive impairment. Further investigation is needed to determine whether the cognitive impairment observed in this study was affected by both age and the high-altitude environment.

This study revealed that the FA value of the body of the corpus callosum in the plain group was negatively correlated with the Logical Memory-Immediately and Logical Memory-Delay scores, whereas no significant correlation was found in the high-altitude group. Logical memory refers to the ability to sequentially encode, store and retrieve plots or information. The Logical Memory test is often used to evaluate verbal memory ability and to assess cognitive function. Previous studies have shown that delayed recall added to a memory task (i.e., WMS-III logical memory, Story A) can increase the overall accuracy of the differentiation between mild cognitive impairment (MCI) and normal aging (Rabin et al., 2009). The brain regions associated with logical memory include the left Heschl gyrus (Dickey et al., 2002), hippocampus (Rhein et al., 2020), cingulate gyrus (Medhi et al., 2021) and cerebellar vermis (Levitt et al., 1999). In addition, abnormalities in the corpus callosum can also cause memory impairment (Zaidel, 1995; Paul et al., 2016). Related studies (Estruch et al., 1997; Ahmed et al., 2012) have shown that abnormalities of the corpus callosum (atrophy, less gray matter, or reduced thickness) are significantly associated with logical memory, visual memory, or visual representation. After partial or total resection of the corpus callosum, all patients developed logical memory impairment (Zaidel and Sperry, 1974). These results indicate that the corpus callosum is also a relevant brain region responsible for logical memory, which is consistent with the results of this study. For the first time, this study explored the correlation between logical memory and the FA value of the body of the corpus callosum. However, no significant correlation was found between the FA value of the body of the corpus callosum and logical memory in the high-altitude group. These findings indicate that the high-altitude environment has not caused logical memory impairment, which may be related to tissue regeneration in other related brain regions responsible for the logical memory changes induced by prolonged high-altitude exposure or abnormal brain functional activity (Ding et al., 2015)caused by a combination of adaptive compensatory effects, but the specific pathophysiological mechanisms involved remain to be studied.

This study also has several limitations. First, owing to sample size limitations, this study mainly observed trends in differences between the groups, and correlation analysis only observed correlations, without multiple comparison correction; therefore, subsequent studies with larger sample sizes are needed to further confirm the results of this study. Second, other factors that may affect the study results, such as lifestyle factors, were not considered in this study. Future studies need to include more Han individuals living on the high-altitude and plains for multigroup control analysis. Finally, this study analyzed only DTI data, and other analyses of related MRI data were not performed. Multimodal MRI studies on the comprehensive effects caused by a high-altitude environment are needed in the future.

## 5 Conclusion

This study investigated the correlations between white matter structural changes and clinical indicators in the high-altitude group and the plain group and conducted a correlation analysis on the changes in cognitive function and brain structure after long-term high-altitude exposure. This study revealed that long-term exposure to a high-altitude environment could cause a series of changes in sleep, emotion, and cognitive function, as well as irreversible damage to the white matter microstructure of the body of the corpus callosum, which is the brain region responsible for logical memory. In this study, the high-altitude group did not develop logical memory impairment, which may be due to the existence of adaptive compensation after long-term high-altitude exposure. The findings of this study suggested that TBSS-based DTI analysis could more objectively locate and quantitatively evaluate white matter microstructural changes in individuals after long-term high-altitude exposure and clarified the impact of a high-altitude environment on cognitive function.

## Data Availability

The raw data supporting the conclusions of this article will be made available by the authors, without undue reservation.

## Glossary

AD: axial diffusivity
BMI: body mass index
CSF: cerebrospinal fluid
DBP: diastolic blood pressure
DST: Digital Span Test
DTI: diffusion tensor imaging
FA: fractional anisotropy
FWE: family-wise error
FOV: field of view
GAD-7: Generalized Anxiety Disorder-7
HAG: high-altitude group
HAPC: high-altitude polycythemia
HbA1c: glycosylated hemoglobin
HCT: hemoglobin
HDL: high density lipoprotein
HGB: hemoglobin
LDL: low density lipoprotein
MCH: mean corpsular hemoglobin
MCHC: mean corpsular hemoglobin concentration
MCI: mild cognitive impairment
MCV: mean corpuscular volume
MD: mean diffusivity
MoCA: Montreal Cognitive Assessment
MMSE: the Mini-Mental State Examination
MNI: Montreal neurological institute
MRI: magnetic resonance imaging
PCV: packed cell volume
PG: plain group
PHQ-9: the Patient Health Questionnaire-9
PSQI: Pittsburgh Sleep Quality Index
QTP: Qinghai-Tibetan plateau
RBC: red blood corpuscle
RD: radial diffusivity
SAS: the Self-rating Anxiety Scale
SBP: systolic blood pressure
SDS: the Self-rating Depression Scale
TBSS: The tract-based spatial statistics
TE: echo time
TG: triglyceride
TR: repetition time
UV: ultraviolet
VLDL: very low density lipoprotein
